# Mir-660 is downregulated in lung cancer patients and its replacement inhibits lung tumorigenesis by targeting MDM2-p53 interaction

**DOI:** 10.1038/cddis.2014.507

**Published:** 2014-12-11

**Authors:** O Fortunato, M Boeri, M Moro, C Verri, M Mensah, D Conte, L Caleca, L Roz, U Pastorino, G Sozzi

**Affiliations:** 1 Tumor Genomics Unit, Department of Experimental Oncology and Molecular Medicine, Fondazione IRCCS Istituto Nazionale dei Tumori, Milan, Italy; 2 Molecular Bases of Genetic Risk and Genetic Testing Unit, Fondazione IRCCS Istituto Nazionale dei Tumori, Milan, Italy; 3 Thoracic Surgery Unit, Fondazione IRCCS Istituto Nazionale dei Tumori, Milan, Italy

## Abstract

Lung cancer represents the leading cause of cancer-related death in developed countries. Despite the advances in diagnostic and therapeutic techniques, the 5-year survival rate remains low. The research for novel therapies directed to biological targets has modified the therapeutic approach, but the frequent engagement of resistance mechanisms and the substantial costs, limit the ability to reduce lung cancer mortality. MicroRNAs (miRNAs) are small noncoding RNAs with known regulatory functions in cancer initiation and progression. In this study we found that mir-660 expression is downregulated in lung tumors compared with adjacent normal tissues and in plasma samples of lung cancer patients with poor prognosis, suggesting a potential functional role of this miRNA in lung tumorigenesis. Transient and stable overexpression of mir-660 using miRNA mimics reduced migration, invasion, and proliferation properties and increased apoptosis in p53 wild-type lung cancer cells (NCI-H460, LT73, and A549). Furthermore, stable overexpression using lentiviral vectors in NCI-H460 and A549 cells inhibited tumor xenograft growth in immunodeficient mice (95 and 50% reduction compared with control, respectively), whereas the effects of mir-660 overexpression were absent in H1299, a lung cancer cell line lacking p53 locus, both in *in vitro* and *in vivo* assays. We identified and validated mouse double minute 2 (MDM2) gene, a key regulator of the expression and function of p53, as a new direct target of mir-660. In addition, mir-660 expression reduced both mRNA and protein expression of MDM2 in all cell lines and stabilized p53 protein levels resulting in an upregulation of p21^WAF1/CIP1^ in p53 wild-type cells. Our finding supports that mir-660 acts as a tumor suppressor miRNA and we suggest the replacement of mir-660 as a new therapeutic approach for p53 wild-type lung cancer treatment.

Lung cancer is the leading cause of cancer death worldwide, resulting in >1.4 million deaths/year.^1^ Lung tumors are often discovered as locally advanced or metastatic disease, and despite improvements in molecular diagnosis and targeted therapies, the overall 5-year survival rate remains in the 10–20% range. Indeed, nonsmall cell lung cancer (NSCLC) is poorly chemosensitive to most of the available agents with response rates ranging from 10 to 25%.^2^ The discovery of recurrent mutations in the epidermal growth factor receptor (EGFR) kinase,^3^ as well as gene fusion products involving the anaplastic lymphoma kinase (ALK),^4^ has led to a marked change in the treatment of patients with lung adenocarcinoma, the most common type of lung cancer.^5, 6^ To date, patients with mutations in the EGFR gene, suitable for targeting by EGFR tyrosine kinase inhibitors, represent roughly 10%, whereas the subgroup of tumors with ALK rearrangements, targeted by ALK inhibitors, is only ~5%.^7^ Thus, the majority of lung tumors lack effective treatment and novel therapeutic strategies are still needed.

MicroRNAs (miRNAs) are short noncoding RNAs, 20–24 nucleotides long, that have important roles in almost all biological pathways,^8, 9, 10, 11^ and influence cancer-relevant processes, such as proliferation,^12^ cell cycle,^13^ apoptosis,^14^ and migration.^15^ Many studies have reported the critical role of miRNAs in lung cancer pathogenesis and their potential as biomarkers for lung cancer risk stratification,^16^ outcome prediction,^17^ and classification of histological subtypes.^18, 19^ miRNAs are actively released by various cell types and can be detected in biological fluids, such as plasma and serum, making them suitable as circulating biomarkers in NSCLC.^20, 21^

There is limited evidence of mir-660 deregulation in cancer and little is known about its role in lung tumorigenesis and its putative target genes. Mir-660 has been reported to be upregulated in chronic lymphocytic leukemia^22, 23^ and in leukemic cells after treatment with 4-hydroxynonenal, a compound that induces differentiation and blocks proliferation of leukemic cells.^24^ In a previous study we demonstrated that mir-660 was one of the 24 miRNAs deregulated in plasma samples of NSCLC patients identified in a low-dose computed tomography (LDCT) screening trial.^20^

The p53 tumor suppressor protein is a key regulator of cell cycle G0/G1 checkpoint, senescence, and apoptosis in response to cellular stress signals.^25, 26^ Mouse double minute 2 (MDM2), a p53–E3 ubiquitin ligase,^27^ is the principal negative regulator of the expression level and function of p53.^28, 29^ Several studies have illustrated different mechanisms of p53 regulation by MDM2,^30, 31^ such as binding transactivation region of p53,^32, 33^ promoting nuclear export and cytoplasmic accumulation of p53 by monoubiquitination,^34, 35^ and inducing p53 proteosomal degradation by polyubiquitination.^36^ In addition, *MDM2* gene has been reported to be amplified or overexpressed in a variety of human cancers, such as sarcoma,^37^ lymphoma,^38^ breast cancer,^39^ lung cancer,^40^ and testicular germ cell tumor.^41^ Several miRNAs targeting MDM2 have been identified, such as the mir-143/mir-145 cluster that can be induced by p53,^42^ as well as mir-25 and mir-32, known to inhibit tumor glioblastoma growth in mouse brain.^43^

In this study, we report that mir-660 is downregulated in tissue and plasma samples of lung cancer patients and demonstrate that mir-660 replacement impairs the functionality of p53 wild-type (wt) lung cancer cells and inhibits *in vitro* and *in vivo* tumor growth. We showed that all the effects observed after mir-660 overexpression were absent in p53 ko cells, identified *MDM2* as mir-660 direct target gene and indicate impairment of the MDM2/p53 interaction as the mechanism underlying tumor growth inhibition.

## Results

### Diagnostic and prognostic value of mir-660 in plasma and tissue samples of lung cancer patients

**Figure 1:**
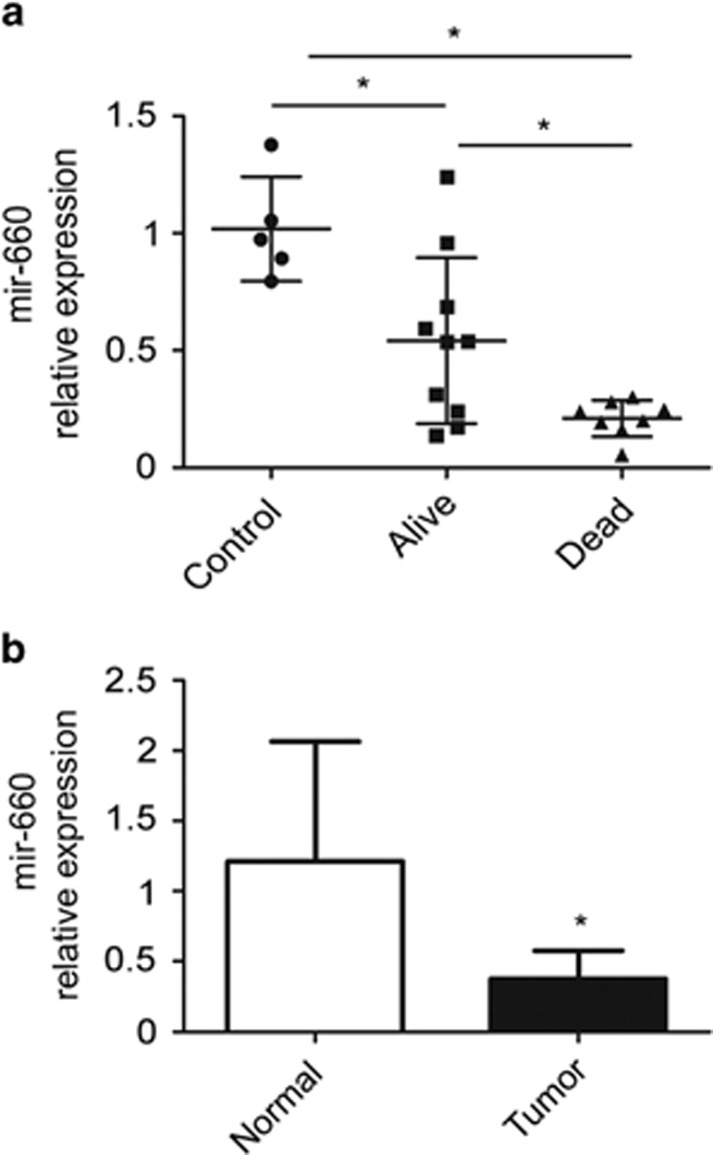
Mir-660 is downregulated in tumor tissue and plasma. (**a**) Dot plots showing mir-660 levels in plasma samples. Data were normalized on the average of each card. *P<0.05 *versus* each group. (**b**) Histogram showing mir-660 expression levels in lung cancers compared with distant normal tissues. *P<0.05 *versus* normal tissues. Data are expressed as mean±S.D.

We performed high-throughput miRNA expression profile of plasma samples from 18 lung cancer patients and 27 matched disease-free individuals grouped in 5 pools collected during the INT-IEO LDCT screening trial^44^(Supplementary Table 1). Among those miRNAs significantly deregulated between patients and controls, we found that mir-660 was progressively downmodulated in patients with good prognosis (alive) (mir-660 relative expression=0.54±0.35 *versus* 1.02±0.22, *P*<0.05), and patients with poor prognosis (dead) (mir-660 relative expression=0.21±0.08 *versus* 1.02±0.22, *P*<0.05) (Figure 1a) compared with disease-free subjects.

To analyze mir-660 expression also in lung tissue samples, 20 pairs of tumor and distant normal lung tissues obtained from lung cancer patients identified in the Multicentric Italian Lung Detection (MILD) trial^45^ were selected (Supplementary Table 1). As reported in Figure 1b, mir-660 expression was significantly reduced in tumor compared with paired normal lung tissues of the patients (mir-660 relative expression=0.38±0.2 *versus* 1.21±0.85, *P*<0.05). Furthermore, we analyzed p53 mutational status in the series of lung cancer patients used for tissue miRNA profiling and we found p53 mutations in 9 out of 20 patients, but the p53 status did not correlate (*P*=0.37) with mir-660 expression levels.

### mir-660 re-expression impairs cancer cell functions

**Figure 2:**
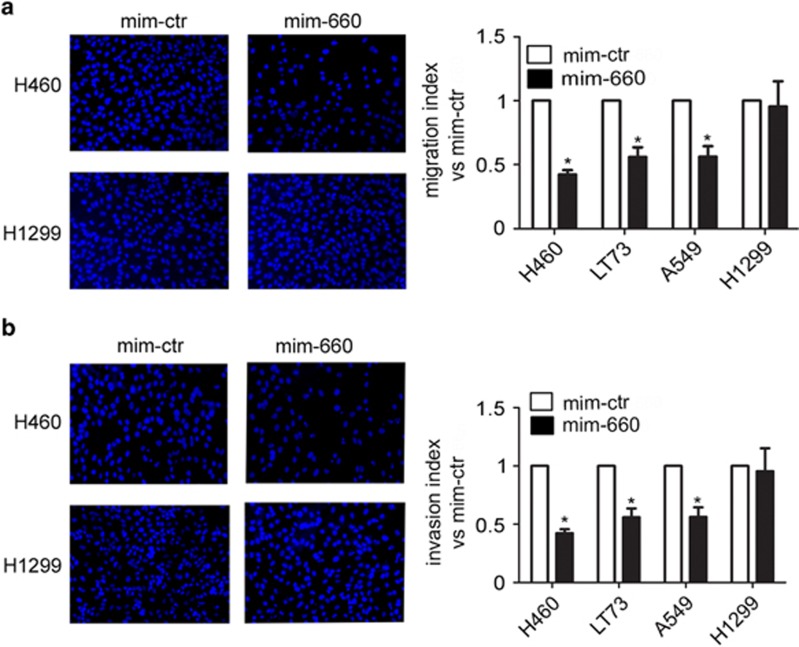
Mir-660 inhibits migration and invasion of lung cancer cells. Mir-660 decreases (**a**) migratory and (**b**) invasive capacity of lung cancer cells in Transwell assay (*n*=5). Representative images of migrated/invaded cells for each condition are shown. Migration and invasion data are expressed as the number of migrated mir-660 overexpressing cells *versus* the number of migrated control cells. All data are expressed as mean±S.E.M. *P<0.05 *versus* cells transfected with control

**Figure 3:**
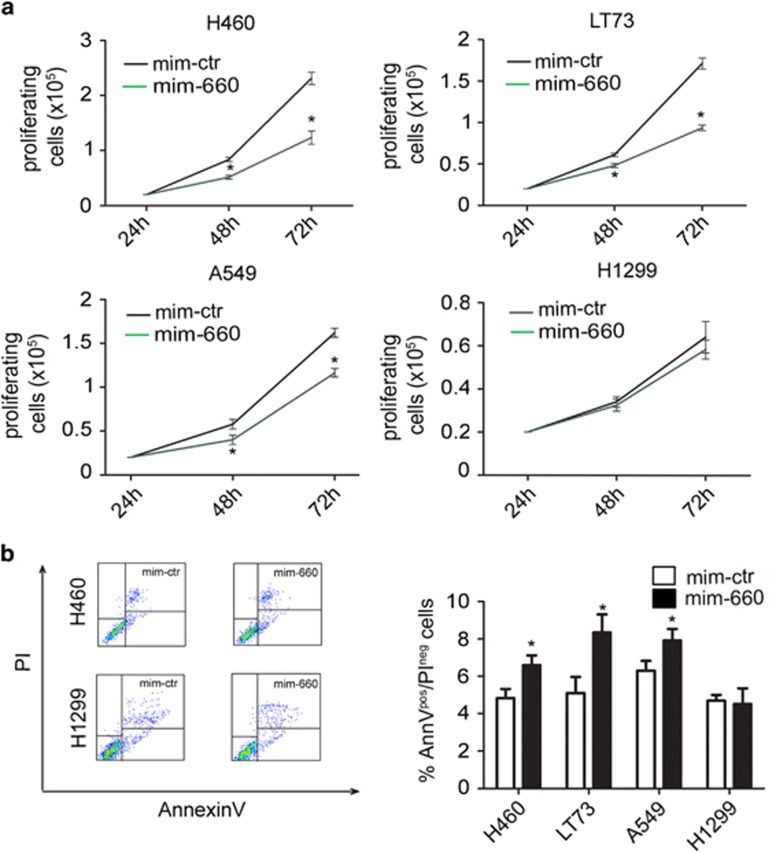
Mir-660 overexpression reduces lung cancer cell growth. (**a**) Cells were transfected with mir-660 or control and viable cells were counted with trypan blue at 72 and 120 h to measure cell growth. Graphs show cell proliferation of mir-660 overexpressing cells compared with control cells. (*n*=5) (**b**) Apoptosis was measured by flow cytometry as annexin V^pos^/PI^neg^ cells (left panel) and graphs show the number of apoptotic cells compared with cell transfected with mimic control (right panel). (*n*=5) All data are expressed as mean±S.E.M. *P<0.05 *versus* cells transfected with control

To understand the functional role of mir-660 in lung tumorigenesis, we performed a series of *in vitro* experiments using commercially available miRNA mimics in four different lung cancer cell lines (NCI-H460, LT73, A549, and H1299). mir-660 overexpression resulted in a significantly decrease of migratory (Figure 2a) and invasive (Figure 2b) capacity of the three p53 wt cancer cell lines, but not of the H1299 lung cancer cell line lacking p53 protein. Furthermore, a reduction in cell proliferation at 72 and 120 h after mir-660 transfection was detected in p53 wt cells only (Figure 3a). To explain the decrease in cell proliferation, we evaluated apoptosis by flow cytometry by measuring the AnnexinV^pos^/PI^neg^ cells in mir-660 transfected cell lines and observed a 20–60% increase in the number of apoptotic cells after 48 h compared with cells transfected with mimic control (Figure 3b). Cell cycle progression analysis also showed a significant increase of G0/G1 cell fractions indicating a cell cycle arrest (Table 1). Interestingly, the absence of these effects in H1299 lacking p53 locus suggested a potential interaction of mir-660 with the p53 pathway.

### MDM2 is a direct target of mir-660

**Figure 4:**
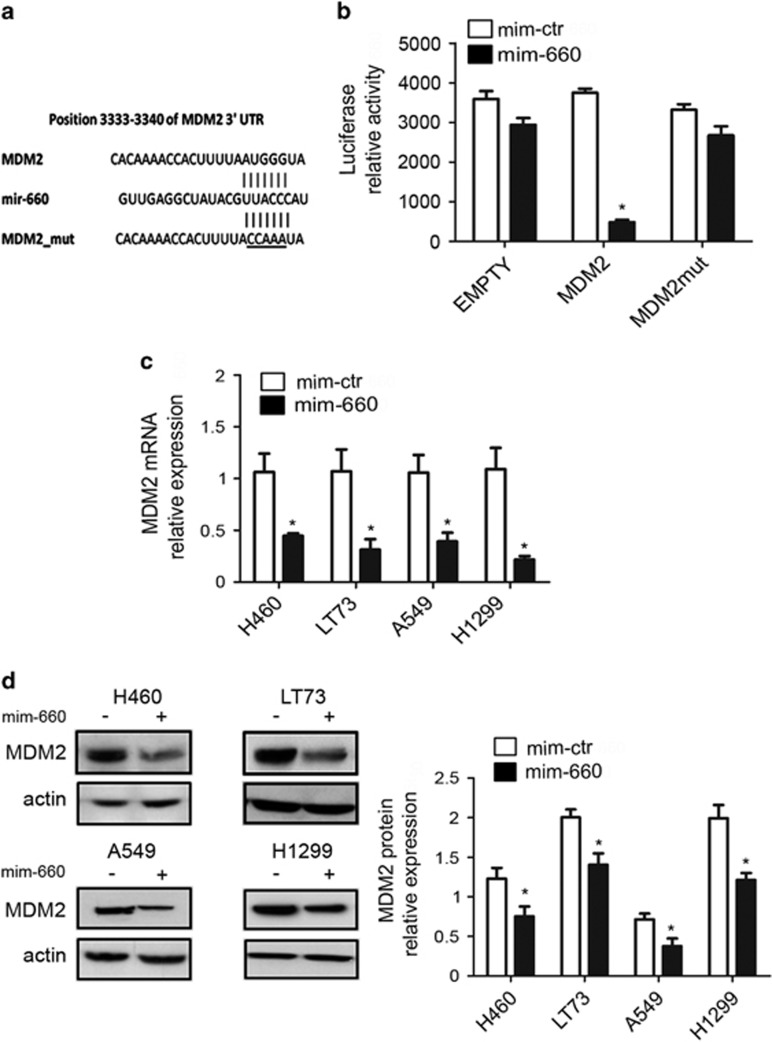
MDM2 is a direct target of mir-660. (**a**) Predicted MDM2 3′ UTR-binding site for mir-660. The alignment shown is of the mir-660 seed sequence with MDM2 3′ UTR. (**b**) Bar graphs showing average luciferase activity. Reporter systems were transfected in HEK293 with MDM2 wt or mutated and EMPTY 3′ UTR in combination with mir-660 mimics or control. (**c**) MDM2 mRNA levels in lung cancer cells transfected with mimic mir-660 or mimic control (*n*=5). (**d**) Results of MDM2 analysis by western blot (*n*=4) and representative western blot bands. All data are expressed as mean±S.E.M. (*P<0.05)

**Figure 5:**
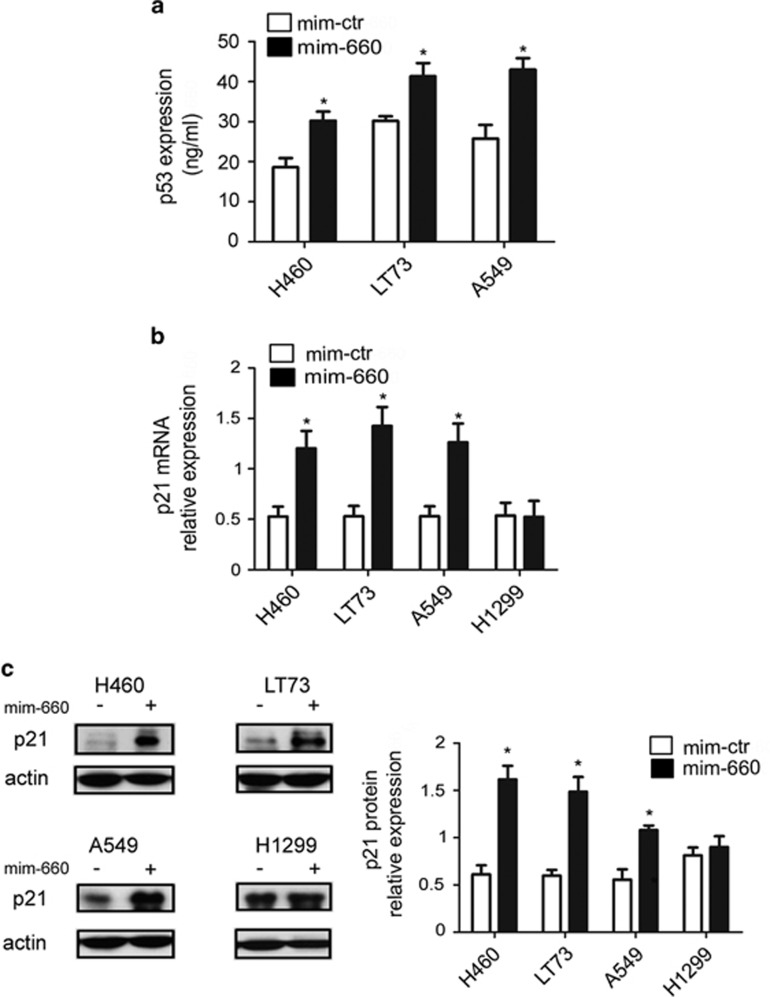
Mir-660 increased p53 levels and function. (**a**) p53 levels after mir-660 overexpression measured by ELISA (*n*=4). (**b**) p21 mRNA levels in lung cancer cells transfected with mimic mir-660 or mimic control (*n*=4). (**c**) p21 expression analysis by western blot (*n*=4) and representative western blot bands for all cell lines. All data are expressed as mean±S.E.M. (*P<0.05)

On the basis of *in vitro* results, we identified, using *in silico* programs, putative mir-660 targets, focusing on those mRNA encoding for proteins that are components of the p53 pathway. The analysis identified the 3′ untranslated region (UTR) of MDM2 as a complementary sequence for the binding of mir-660 (Figure 4a); MDM2 is an E3 ubiquitin–protein ligase with a central role is the physiological regulation of p53 levels. To prove that MDM2 is a direct target of mir-660, we performed a luciferase reporter assay using commercial custom made 3′ UTR MDM2 and observed a strong downmodulation (87% reduction) of the luciferase activity when HEK-293 cells were cotransfected with mir-660 (Figure 4b). Target specificity was validated either using a 3′ UTR EMPTY vector or by site-directed mutagenesis in the putative mir-binding sites where we did not detect any change in luciferase activity (Figure 4b). We also observed a significant reduction of MDM2 mRNA 72 h after mir-660 transfection as measured by real-time PCR (60% reduction in NCI-H460, 70% in LT73 and 63% in A549 compared with control) (Figure 4c) and a decreased MDM2 protein expression by Western Blot in all tested cell lines (39% decrease in NCI-H460, 30% in LT73, and 47% in A549 compared with control) (Figure 4d). Furthermore, to confirm that mir-660-induced reduction of MDM2 expression affects p53 protein we measured p53 levels on cells lysates and a significant increase of p53 protein expression in all p53 wt cell lines was detected (63% increase in NCI-H460, 37% in LT73, and 67% in A549 compared with control) (Figure 5a).

To demonstrate that the antitumoral activity of mir-660 is p53 dependent, we analyzed mRNA levels of p21^WAF1/CIP1^, a cyclin-dependent kinase inhibitor that functions as p53-dependent cell cycle checkpoint, and observed a significant increase of p21 levels after mir-660 overexpression (2.3-fold increase in NCI-H460, 2.7 in LT73, and 2.4 in A549 compared with control) (Figure 5b). According to p21^WAF1/CIP1^ mRNA level upregulation, a western blot analysis on cell lysates showed a comparable increase of p21^WAF1/CIP1^ protein levels (2.6-fold increase in NCI-H460, 2.5 in LT73, and 1.7 in A549 compared with control) (Figure 5c).

Interestingly, downmodulation of MDM2 was visible also in H1299 p53-null cells (40% reduction) without stimulation of p21^WAF1/CIP1^ transcription or protein expression indicating that the presence of a functional p53 protein is fundamental for mir-660 to exert antitumoral effects through the regulation of MDM2 levels.

### Mir-660 stable overexpression has tumor suppressive effects *in vitro*

To obtain a stable mir-660 overexpression in all cell lines used, we created stable mir-660 transfectants using lentiviral vectors (Supplementary Figures 1A and C). Furthermore, to confirm mir-660 antitumoral activity we performed *in vitro* assays using stable mir-660 overexpressing cells and we observed a decrease in migratory (Supplementary Figure 2A) and invasive (Supplementary Figure 2B) ability of these cells and a reduction in cell proliferation compared with control (Supplementary Figure 2C). Stable mir-660 overexpression induced a significant increase of apoptotic cells measured as the AnnexinV^pos^/PI^neg^ in NCI-H460 and A549 cells (2.5-fold increase in NCI-H460 and 1.8 in A549 compared with control) (Supplementary Figure 2D). According to data obtained with transient transfection in H1299 cells these effects were totally abrogated. Interestingly, cell cycle analysis showed a marked increase of apoptotic cells (subG0) and a strong G0/G1 arrest in NCI-H460 and A549, respectively, whereas no differences were observed in H1299 p53-null cells (Supplementary Figure 2E and Supplementary Table 2). In all cell lines, stable mir-660 overexpression reduced MDM2 protein levels as shown by western blot analysis (48% protein reduction in NCI-H460, 35% in A549, and 45% in H1299 compared with control) (Supplementary Figure 2F). Unfortunately, stable mir-660 transfectants of LT73 cells could not be obtained likely owing to the toxicity of GFP reporter gene in this primary established cell line.

### Mir-660 inhibits xenograft tumor growth

Prompted by the findings of mir-660 downregulation in lung cancer patient tissues and plasma and by the antitumoral effects observed after mir-660 overexpression in *in vitro* assays, we evaluated the potential role of this miRNA in the inhibition of tumor growth in immunodeficient mice.

Subcutaneous injection of mir-660 transiently transfected p53 wt NCI-H460 (Supplementary Figure 3A) and A549 (Supplementary Figure 3B) cells in nude mice resulted in a slight initial delay in tumor growth. After this initial effect (10–15 days for NCI-H460 and 30–35 days for A549), tumors restarted to grow at rates comparable to control transfected cells. LT73 transiently transfected cells showed a growth delay at 30–35 days compared with control (data not shown). On the other hand, in p53-null H1299 cells (Supplementary Figure 3C), transfection of mir-660 had no effects on xenograft growth.

**Figure 6:**
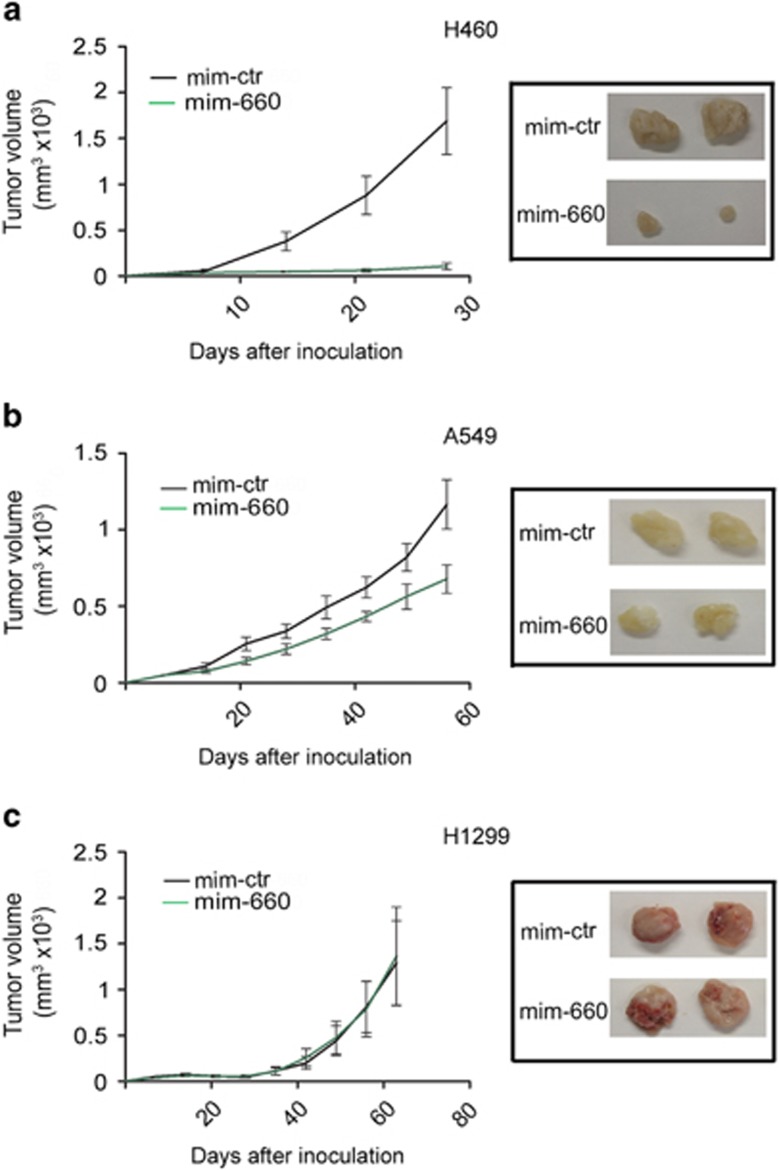
Mir-660 inhibited xenograft tumor growth in mice. Graphs show tumor growth of mir-660 overexpressing cells s.c. injected in both flanks of nude mice compared with control (*n* =5 per group). MiRNAs were stable transfected in (**a**) NCI-H460, (**b**) A549, and (**c**) H1299. All data are expressed as mean±S.E.M. (*P<0.05 *versus* mim-ctr). Representative images of tumor size for each condition (right panels)

Mir-660 expression levels returned similar to those of control cells 20 days after cell transfection (Supplementary Figure 3D), suggesting a correlation between mir-660 transient overexpression and the initial delay in tumor growth observed in p53 wt cell lines xenografts. Indeed, injections of stable transfectants of mir-660 led to a complete *in vivo* growth inhibition (95% reduction compared with control) in NCI-H460 cells (p53 wt) (Figure 6a). These effects were less pronounced in A549 (50% inhibition) and completely absent in H1299 transfected cells (Figures 6b and c), lacking the MDM2-negative regulators p14^arf^ and p53 protein, respectively. These results highlight the central role of the MDM2/p53 pathway in mir-660 mediated effects, also in *in vivo* xenograft models.

## Discussion

MiRNAs negatively regulate gene and protein expression by acting as oncogenes or tumor suppressors and are involved in the pathogenesis of lung diseases including lung cancer. The rationale of using miRNA as therapeutics agents in lung cancer management is based on the assumption that miRNAs have an important role in lung development,^46^ their expression levels are deregulated in lung cancer patients compared with healthy subjects^17^ and that modulation of miRNA expression, both *in vitro* and *in vivo*, can modify the cancer phenotype.^47, 48^

Different strategies of miRNAs therapeutics can be envisaged according to the expression status of miRNAs in the tumor: inhibition of oncomirs or overexpression of tumor suppressor miRNAs.^49^

In this study, starting from the finding that mir-660 levels are downmodulated in plasma of lung cancer patients and inversely correlated with prognosis and that mir-660 expression was significantly downregulated in lung tumors compared with normal lung tissues, we explored the functional role of mir-660 in lung tumorigenesis.

Upon mir-660 replacement, both in transient or in stable transfections, we showed a tumor growth inhibition effect, *in vitro* and *in vivo*, likely mediated by mir-660-induced impairment of the MDM2/p53 interaction. The transcription factor p53 is expressed at low concentration in normal cells and it has an important role in cell cycle regulation.^50^ In physiological condition, p53 levels are suppressed by the activity of MDM2. Disruption of the p53–MDM2 interaction is the pivotal event for p53 activation, leading to p53 stabilization and its biological functions, such as cell growth control, apoptosis, and modulation of cell migration.^51, 52^

Mir-660 overexpression led to arrest of proliferation in G0/G1 checkpoint and induction of apoptosis in a p53-dependent manner.^53^ Indeed, these effects were achieved by *in vitro* replacement of mir-660 in p53 wt NCI-H460 and A549 cells, whereas in H1299 p53-null cells no effects were appreciable on cell cycle or on apoptosis even if a decrease of MDM2 expression levels was detected. We showed that mir-660 induced p53 stabilization and increased its transcriptional activity resulting in an upregulation of its target gene, p21^WAF1/CIP1^, which regulates cell cycle through inhibition of cyclin-dependent kinases required for progression from G1 to S phase and it is also involved in the apopotic process.

Similar results were obtained in *in vivo* experiments where a significant inhibition of tumor xenograft growth was obtained with mir-660 stable transfection of NCI-H460 and A549 cells and not of p53-null H1299 cells.

Several studies indicate that p53 tumor suppressor activity is frequently inactivated in NSCLC patients by mutations (53% of all lung cancer)^7, 54, 55^ or by interaction with MDM2, which eliminates wt p53.^56^ MDM2 amplification occurs in 7% of human tumors^57^ with varying degrees of amplification between tumor types, such as liposarcoma (50–90%), osteosarcomas (16%), esophageal carcinomas (13%), and NSCLC (6%). Notably, MDM2 amplification and p53 mutations are essentially mutually exclusive^58^ and, in the past few years, small-molecule antagonists of p53–MDM2 interaction as nutlins^59, 60^ or MDM2 inhibitors^61^ have been developed.

These observations suggest that reconstitution of p53-dependent pathways in tumor cells is an effective therapeutic strategy^62^ and restoration of p53 activity using mir-660 represents an attractive approach for lung cancer therapy. The principal advantage of using miRNAs as therapeutic agent is that they could target several genes of redundant pathways and thus potentially able to achieve a broad silencing of protumoral pathways. A very preliminary bioinformatic analysis revealed that mir-660 potentially targets several transcription factors, proteases and other regulators of cell growth and survival. Interestingly, we showed that relatively small changes in the expression of miRNA and its target gene could induce relevant phenotypic alterations of lung cancer cells, both *in vitro* and *in vivo*.

Our results provide evidence that mir-660 behaves as a tumor suppressor miRNA in lung cancer and that mir-660 replacement could represent a potential nontoxic successful therapy for a large subset of lung cancer patients where p53 locus is not genetically altered by mutation or deletion.

## Materials and Methods

### Population study

Tissue and plasma samples were collected from high-risk heavy smoker volunteers aged from 50 to 75 years old including current or former smokers with a minimum pack/year index of 20 enrolled in 2 independent LDCT screening trials performed at our Institution. For miRNA analysis, lung tissue samples from 20 lung cancer patients from the MILD trial were selected; in addition, plasma samples from 18 lung cancer patients and 27 disease-free individuals from the Istituto Nazionale dei Tumori – Istituto Europeo di Oncologia (INT-IEO) trial were selected for miRNA analysis (Supplementary Table 1).^44, 45^

### MiRNA expression analysis

For plasma samples, total RNA was isolated from 200 *μ*l of plasma using the *mir*Vana PARIS kit (Thermo Fisher Scientific, Waltham, MA, USA) according to the manufacturer's instructions and eluted in 50 *μ*l of elution buffer. High-throughput analyses were performed using the Megaplex Pools Protocol on microfluidic card type A (Thermo Fisher Scientific) as previously described.^20^ For plasma analysis, data were normalized on the average of each card.

For tissue samples, total RNA was extracted using Trizol (Thermo Fisher Scientific) following manufacturer's instructions and quantified using the NanoDrop 2000 (Thermo Fisher Scientific).

For cultured cells, total RNA was isolated using *mir*Vana PARIS Kit (Thermo Fisher Scientific) following the manufacturer's instructions. Reverse transcription was performed using the TaqMan microRNA Reverse Transcription Kit according to the manufacturer's instruction (Thermo Fisher Scientific). MiRNA expression was analyzed by the Applied Biosystems 7900 System (Applied Biosystems, Foster City, CA, USA) and normalized to the small nucleolar RNU6B for tissues and RNU48 for cells. For gene expression analysis, cDNA synthesis was performed using 250 ng of total RNA. The relative quantification of the all analyzed genes was performed using ready-to-use Assay-on-Demand (Thermo Fisher Scientific), and human GAPDH was used as endogenous control for normalization.

### Cell lines and miRNA transfection

Human lung cancer cell lines, NCI-H460, A549, and H1299, were obtained from the American Type Culture Collection (ATCC). LT73 cells were derived in our laboratory from a primary lung tumor of a 68–year-old Caucasian male with lung adenocarcinoma. Cells were cultured in RPMI 1640 (Gibco, Thermo Fisher Scientific) medium supplemented with 10% heat inactivated fetal bovine serum (FBS) and 1% penicillin–streptomycin (Sigma-Aldrich, St. Louis, MO, USA). Cells were transfected using *mir*Vana miRNA mimics using Lipofectamine 2000 (Thermo Fisher Scientific) according to the manufacturer's instructions (Supplementary Figure 1A and C).

### Proliferation assay

For proliferation assay cells were seeded in a 12-well plate at 2 × 10^5^ cells for A549, H1299 and LT73, and 1.5 × 10^5^ cells for NCI-H460. Viable cells were counted after 72 and 120 h by trypan blue (Sigma-Aldrich). Each experiment was performed in triplicates.

### Migration and invasion assay

For migration assay 10^5^ cells were plated on the top chamber of FluoroBlok Cell Culture Inserts (BD Biosciences, San Diego, CA, USA). RPMI plus 10% FBS was added to the bottom chamber and incubated at 37 °C and 5% CO_2_. For the invasion assay FluoroBlok Cell Culture Inserts were coated with matrigel (BD Biosciences). After 24 h, cells that had migrated to the bottom side of the insert were fixed and stained with DAPI. Migrated cells were counted using fluorescence microscopy. Migration and invasion data are expressed as the number of migrated mir-660 overexpressing cells *versus* the number of migrated control cells.

### Apoptosis evaluation

Apoptosis was measured by quantifying the percentage of Annexin V^pos^/Propidium Iodide^neg^ cells by flow cytometry. The percentage of apoptotic cells was evaluated 48 h after miRNA transfection using the Annexin V Kit (Milteniy Biotec, Auburn, CA, USA) according to the manufacturer's protocol.

### Cell cycle evaluation

Cells were fixed with 70% cold ethanol and stained with propidium iodide (50 *μ*g/ml) for 40 min. Cells were analyzed by flow cytometry using BD FACS Calibur and Cell Quest software (BD Biosciences).

### Western blot analysis

Proteins were extracted by incubation with RIPA buffer and quantified by Bradford reagent. Twenty-five micrograms of protein were separated on Nupage 4–12% polyacrylamide gels (Thermo Fisher Scientific) and transferred to polyvinylidene difluoride membranes (PVDF, GE Healthcare Life Science, Piscataway, NJ, USA) to be probed with the following antibodies: mouse anti-MDM2 (1 : 500, Abcam, Cambridge, UK); mouse anti-p21 (1 : 1000, Cell Signaling, Danvers, MA, USA), and rabbit anti-*β*-actin (1 : 5000, Sigma-Aldrich). For detection, goat anti-rabbit or goat anti-mouse secondary antibodies conjugated to horseradish peroxidase (1 : 2000, GE Healthcare Life Science) were used. Signal detection was performed via chemiluminescence reaction (ECL, GE Healthcare Life Science). WB quantification was performed using ImageJ software analysis (National Institutes of Health, Bethesda, MD, USA).

### p53 ELISA

P53 protein levels in cancer cells lysates were measured using p53 Human ELISA kit (Abcam) according to manufacturer's instructions.

### Luciferase assays

To investigate whether MDM2 is a direct target of mir-660, the 3′ UTR of MDM2 was purchased from Switchgear Genomics (Menlo Park, CA, USA). Conserved binding sites in MDM2 3′ UTR at position 3333–3340 was identified using TargetScan (http://www.targetscan.org). An empty vector was used as control. Furthermore, the predicted target site for miR-660 was mutated by direct mutagenesis of the pLightSwitch_MDM2 3′ UTR vector, using the PCR-based QuikChange II XL site-directed mutagenesis kit (Stratagene, La Jolla, CA, USA) according to the manufacturer's instructions and the following primers: Fw 5′-CAAAACCACTTTTA*CCAAA*TACAGAGTTAAATTTG-3′ Rev 5′-CAAATTTAACTCTGTA*TTTGG*TAAAAGTGGTTTTG-3′ (mutated nucleotides are italicized). The presence of the mutations was confirmed by sequencing. The different luciferase constructs were transfected into HEK293 cells together with miR-660 or a scrambled oligonucleotide sequence (control). Cells were cultured for 48 h and assayed with the Luciferase Reporter Assay System (Switchgear Genomics).

### Generation of stable mir-660 overexpressing cells

To obtain stable mir-660 overexpressing cells we performed experiments using SMARTchoice lentiviral vector (Thermo Fisher Scientific). Lung cancer cells were seeded at 5 × 10^4^ in each well of 24-well plates and infected with mir-660 or control lentiviral vector at the multiplicity of infection of 10 (10 infectious units for each target cells). After 72 h of infection, cells were selected with Puromycin and miRNA overexpression was quantified at 10 and 30 days post infection (Supplementary Figure 1).

### *In vivo* assays

Animal studies were performed according to the Ethics Committee for Animal Experimentation of the Fondazione IRCCS Istituto Nazionale Tumori, according to institutional guidelines previously described.^63^ All experiments were carried out with female CD-1 nude mice or SCID mice, 7–10 weeks (Charles River Laboratories, Calco, Italy). Mice were maintained in laminar flow rooms, with constant temperature and humidity and had free access to food and water.

Lung cancer cells, transfected with mimic-660 or control, were harvested and resuspended in Matrigel/RPMI (1 : 1). 5 × 10^5^ cells were injected subcutaneously in the flanks of 4–6-week-old female nude mice. For each groups five mice were used and injections were performed in two flanks of each animal (*n*=10 tumors/group). Xenograft growth was measured weekly using a calliper.

### Statistical analysis

Statistical significance was determined with unpaired or paired *t-*tests. *P*-values <0.05 were considered statistically significant.

### Author contributions

OF, MB, LR, UP and GS designed the research; OF, MB, MM, CV, MM, DC and LC performed the research; OF, MB, MM, CV, LC, LR and GS analyzed the data; OF, MB and GS wrote the paper; GS and UP gave the study supervision. All authors participated in the critical revision of the report

## Figures and Tables

**Table 1:** Transient mir-660 overexpression induced G0/G1 cell cycle arrest

	**% G0/G1 cells**	***P*-value**	**% S cells**	**% G2/M cells**
*NCI-H460*				
Mim-ctr	77.9±1.5	<0.01	12.1±1.6	7.4±2.0
Mim-660	83.8±1.0	—	10.8±1.0	3.0±1.3
				
*LT73*				
Mim-ctr	66.1±1.7	0.01	16.7±3.8	15.6±1.6
Mim-660	72.7±1.7	—	14.1±1.0	12.0±1.3
				
*A549*				
Mim-ctr	56,5±3.2	0.03	19,9±1.3	15.0±3.0
Mim-660	62,6±2.3	—	18.8±1.2	12.9±3.2
				
*H1299*				
Mim-ctr	81.9±5	0.40	8.2±3.2	6.1±2.5
Mim-660	83.8±3.1	—	7.5±1.3	5.4±1

All data are expressed as mean±S.E.M. (*n*=5, *P<0.05 *versus* mim-ctr)

## Abbreviations

3′ UTR: 3′ untranslated region
ADC: adenocarcinoma
ALK: anaplastic lymphoma kinase
EDTA: ethylenediaminetetraacetic acid
EGFR: epidermal growth factor
FACS: fluorescence-activated cell sorter
FBS: Fetal Bovine Serum
INT-IEO: Istituto Nazionale dei Tumori—Istituto Europeo Oncologia
LDCT: low-dose computed tomography
MDM2: mouse double minute 2
MILD: multicentric Italian lung detection
Mim: microRNA-mimic
miRNA: microRNA
NSCLC: non small cell lung cancer
PCR: Polymerase Chain Reaction
SCC: squamous cell carcinoma
WT: wild type

## Notes

Gabriella Sozzi, Mattia Boeri, and Ugo Pastorino are coinventors for one Italian patent application (number: 1406672) granted the 03/07/2014 in relation to the work described. The remaining authors declare no conflict of interest.
