# Supplementary figures and images for: Mir-660 is downregulated in lung cancer patients and its replacement inhibits lung tumorigenesis by targeting MDM2-p53 interaction
Source: Cell Death Dis. 2014 Dec 11;5(12):e1564–. doi: 10.1038/cddis.2014.507 (PMC4454158; doi:10.1038/cddis.2014.507)

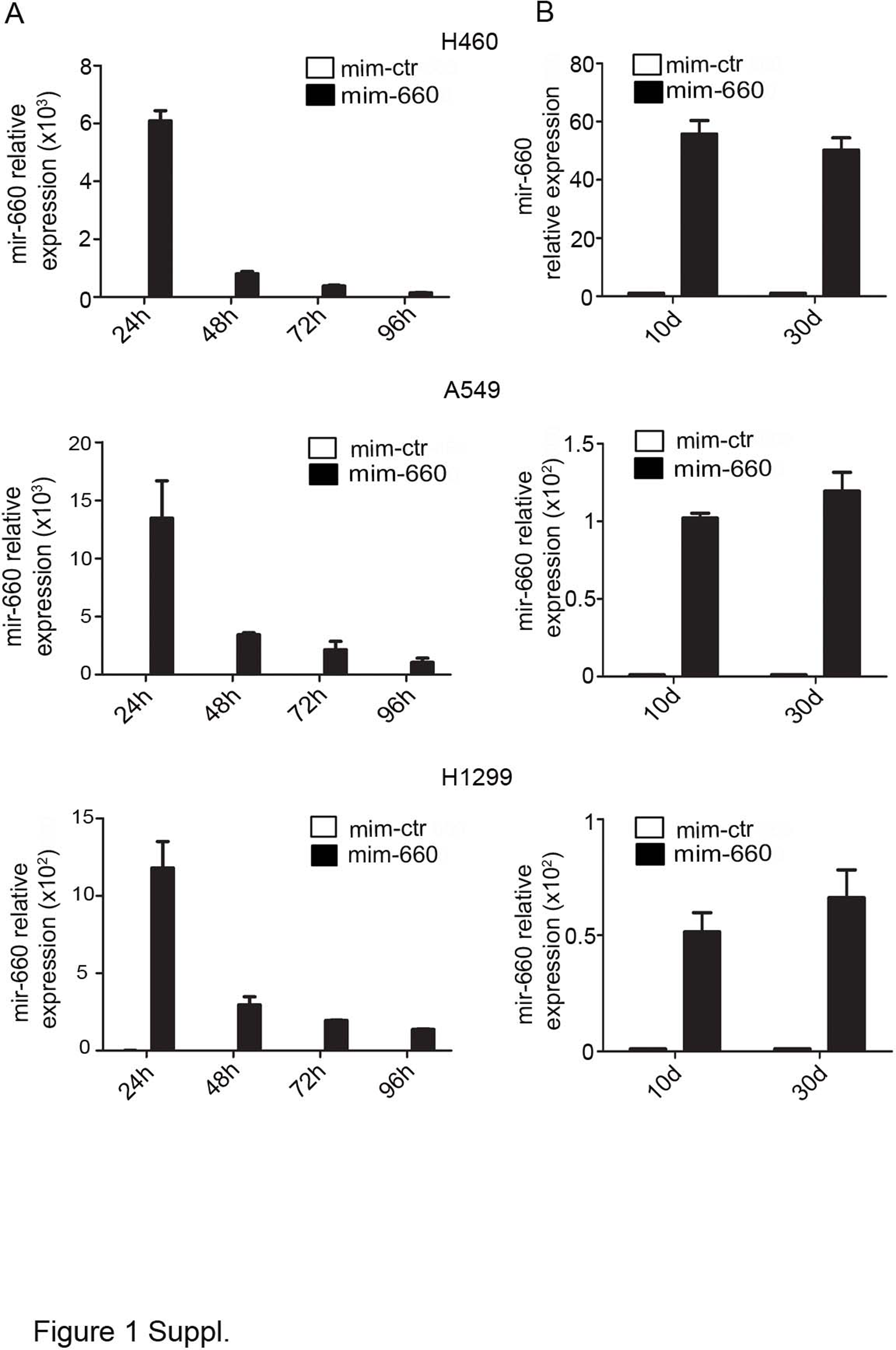

Supplement: Supplementary Figure 1 [file cddis2014507x1.tif]

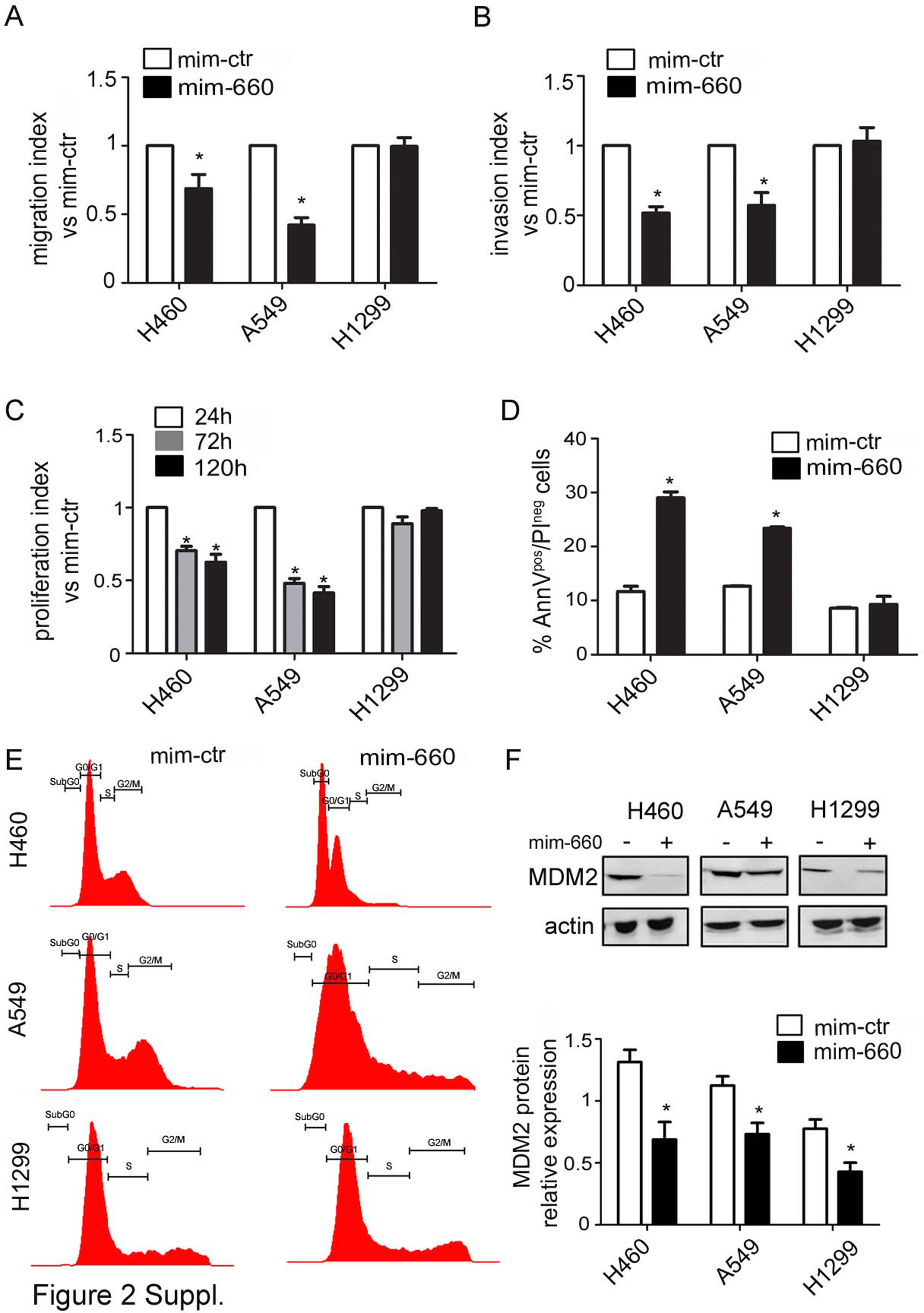

Supplement: Supplementary Figure 2 [file cddis2014507x2.tif]

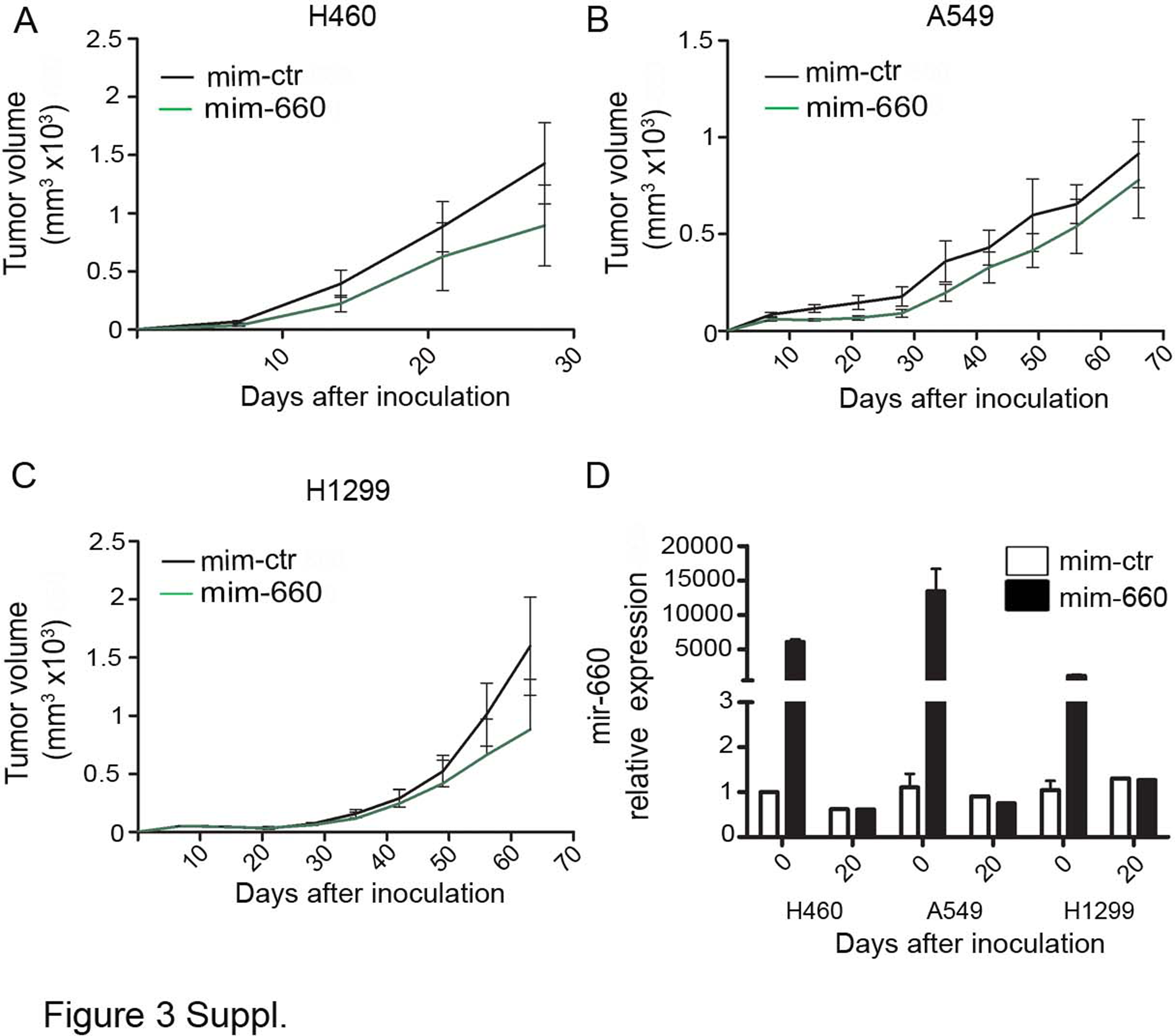

Supplement: Supplementary Figure 3 [file cddis2014507x3.tif]
