# Supplementary material for: Mir-660 is downregulated in lung cancer patients and its replacement inhibits lung tumorigenesis by targeting MDM2-p53 interaction
Source: Cell Death Dis. 2014 Dec 11;5(12):e1564–. doi: 10.1038/cddis.2014.507 (PMC4454158; doi:10.1038/cddis.2014.507)
Supplement: Supplementary Tables [file cddis2014507x4.doc]

**Suppl. Figure 1**. **Mir-660 efficient expression manipulation in human lung cancer lines.** **A)** Relative expression of mir-660 after transient transfection with mir-660 mimic or control in three different cell lines: NCI-H460 (**upper panel**), A549 (**middle panel**) and H1299 (**lower panel**) **B)** Relative expression of mir-660 after stable transfection with mir-660 or control lentiviral vector in three different cell lines: NCI-H460 (**upper panel**), A549 (**middle panel**) and H1299 (**lower panel**) 10 or 30 days post infection. All data are expressed as mean±SEM. (n=3, *p<0.05 vs. mim-ctr).

**Suppl Figure 2. Stable mir-660 expression reduced p53 wt cancer cell functionality.** Stable mir-660 over-expression decreases **A)** migratory and **B)** invasive capacity of lung cancer cells in transwell assay (n=3). **C)** Viable cells were counted with trypan blue at 72 and 120 hours to measure cell growth. Graphs show the proliferation reduction of mir-660 over-expressing cells compared to control cells. (n=3) **D)** Apoptosis was measured by flow citometry ad annexin V^pos^/PI^neg^ cells and expressed as number of apoptotic cells compared to control. (n=3) **E)** Representative graphs of cell cycle analysis in stable mir-660 over-expressing cells compared to controls. **F)** Results of MDM2 analysis by Western blot (n=3) and representative Western blot bands. All data are expressed as mean ± SEM. *p<0.05 vs. mir-660 cells with control.

**Suppl Figure 3.** **Transient mir-660 over-expression delay tumor growth in mice**. Graphs show tumor growth of mir-660 over-expressing cells s.c. injected in both flanks of nude mice compared to control (n=5 per group). MiRNAs were transiently transfected in **A**)NCI-H460, **B**)A549 and **C**)H1299 **D**) Relative expression of mir-660 after transient transfection with mir-660 mimic or control in mice tumors. All data are expressed as mean ± SEM. (*p<0.05 vs. mim-ctr)

±SEM. (n=5, *p<0.05 vs. mim-ctr)

Suppl. Table 1. Characteristics of enrolled subjects

|  | **Trial INT-IEO**  **(n=18)** | **Trial MILD**  **(n=20)** |
| --- | --- | --- |
| **Gender** |  |  |
| Male | 12 (66.7%) | 15 (75.0%) |
| Female | 6 (33.3%) | 5 (25%) |
| **Age** (years) | 58.4 ± 5.4 | 61.7 ± 6.6 |
| **Smoking habit** (Pack-Year index) | 60.8 ± 23.2 | 55 ± 19.8 |
| **Histotype** |  |  |
| ADC | 14 (77.8%) | 14 (70.0%) |
| SCC | 3 (16.7%) | 5 (25.0%) |
| other | 1 (5.5%) | 1 (5.0%) |
| **Stage** |  |  |
| Ia-Ib | 11 (61.1%) | 14 (70.0%) |
| II-III-IV | 7 (38.9%) | 6 (30.0%) |
| **Median Follow up** (months) | 66 | 26 |
| **Prognosis** |  |  |
| Disease free | 10 (55.6%) | 14(70.0%) |
| Alive with disease | 0 | 1 (5.0%) |
| Dead | 8 (44.4%) | 5 (25.0%) |

Suppl. Table 2. Stable mir-660 over-expression impaired cell cycle in p53 wt cells

|  | | **% G0/G1 cells** | **% S cells** | **% G2/M cells** | **subG0** |
| --- | --- | --- | --- | --- | --- |
| **NCI-460** | **Ctr** | 61.6+0.4 | 24.5+0.2 | 9.3+1.7 | 4.5+1.5 |
|  | **660** | 36.4+0.2 | 7.9+0.2 | 3.4+0.1 | 52.4+0.3 |
| **A549** | **Ctr** | 50.6+0.3 | 23.3+1.2 | 25.9+0.9 | 0.2+0.06 |
|  | **660** | 72.5+2.9 | 16.5+1.1 | 10.6+1.8 | 0.3+0.2 |
| **H1299** | **Ctr** | 72.9+1.5 | 19.1+1.8 | 6.1+0.4 | 1.9+1.6 |
|  | **660** | 76.7+2.0 | 15.6+0.8 | 7.1+1.2 | 0.6+0.3 |

All data are expressed as mean±SEM. (n=3, *p<0.05 vs. mim-ctr)
